# The role of C1 inhibitor and complement as acute phase reactants: are we missing the diagnosis of hereditary angioedema?

**DOI:** 10.1186/s13223-021-00607-5

**Published:** 2021-10-09

**Authors:** Peter Stepaniuk, Ana-Maria Bosonea, Persia Pourshahnazari, Adrienne Roos, Amin Kanani

**Affiliations:** 1 Division of Allergy and Immunology, Department of Medicine, University of British Columbia, Vancouver, BC Canada; 2 Allergy and Immunology, Internal Medicine, Edmonton, AB Canada; 3 Division of Hematology/BMT, Department of Medicine, University of British Columbia, Vancouver, BC Canada

**Keywords:** Hereditary angioedema, Acute phase, C1 inhibitor, C4, Reactant, Infection, Illness, CRP, Complement

## Abstract

**Background:**

C1 inhibitor (C1-INH) and complement 4 (C4) have historically been referred to as positive acute phase reactants, however this has never been evaluated in hereditary angioedema (HAE) patients. Low function of C1-INH and low levels of C4 are important in the diagnosis of HAE type 1 and 2. If C1-INH and/or C4 are significant acute phase reactants, their levels may be falsely “normal” in patients with HAE when measured during times of infection or inflammation resulting in missed or delayed diagnosis.

**Case presentation:**

We present a case series of four HAE patients who had C4, C1-INH, c-reactive protein (CRP) and ferritin measured at baseline and again during a self-reported upper respiratory tract infection (URTI) or flu-like illness. We did not identify any HAE patients who had a significant change in their C1-INH functional level in the context of a mild infection. However, the C4 level did increase into the normal range on three occasions (2 patients, with 1 patient having elevation during two separate illnesses).

**Conclusions:**

C1 inhibitor may not be a clinically significant acute phase protein and appears to still be a reliable diagnostic marker of hereditary angioedema, even in times of modest acute inflammation, unlike complement C4 which can be elevated in this setting.

## Background

Hereditary angioedema type 1/2 (HAE) is a rare condition that causes intermittent, acute and potentially life threatening swelling, often precipitated by one of several known triggers [1]. It arises from low function of C1 inhibitor (C1-INH), a complement protein that plays a role in regulating the contact/kalikrein system, coagulation, and classic complement pathways [1–3]. Historically, it has been referred to as a positive acute phase reactant, stimulated by pro-inflammatory cytokines to help fight inflammation or infection [4–6]. The implications of C1-INH and C4 as acute phase reactants in HAE patients, has not been explored to date. If C1-INH or C4 act as significant acute phase reactants, their levels may be falsely “normal” in patients with HAE when measured during times of infection or inflammation. This has significant implications as a low level or function of C1-INH is a critical component in the diagnosis of HAE, and missed or delayed diagnoses of HAE could potentially lead to significant morbidity or even mortality. Complement 4 (C4) is also decreased in HAE and is often used as a screening test for HAE if C1-INH quantitative or functional assay is not readily available. The goal of our case series was to measure C1-INH and C4 in HAE patients during times of modest inflammation, such as during an upper respiratory tract infection (URTI) or flu-like illness, to observe if any significant variation exists compared to their baseline state.

## Case presentation

This prospective case series was conducted in a private allergy practice in Vancouver, British Columbia, Canada from 2017 to 2020. Patients were considered eligible for inclusion if they were greater than 1 year of age and had a confirmed diagnosis of HAE based on a clinical history of angioedema and prior abnormally low C1-INH functional testing (based on Siemens chromogenic assay) on at least two separate occasions. Individuals with other forms of angioedema such as acquired angioedema, ACE-inhibitor associated angioedema or hereditary angioedema with normal C1 inhibitor were excluded. Patients on any prophylactic therapy including C1-INH replacement, androgens or tranexamic acid were also excluded. Each patient had baseline laboratory investigations measuring serum ferritin, c-reactive protein (CRP), C4 and C1-INH function. Additional laboratory requisitions and survey sheets were provided to the patients. When patients developed symptoms suggestive of an URTI or flu-like illness, they were advised to present to the nearest laboratory within 72 h of symptom onset for repeat measurement of ferritin, CRP, C4 and C1-INH function. To be considered symptomatic, patients were required to self-report at least two of the following: fever greater than 38 °C, chills, muscle aches, new cough, sore throat, rhinorrhea, sinus congestion or dyspnea. Patients were also advised to complete and return a patient survey outlining the details of their illness including specific symptoms experienced, duration of illness, severity of illness (1  =  mild symptoms, able to function; 2  =  moderate symptoms, able to function; 3  =  severe symptoms, able to function; 4  =  severe symptoms, difficulty functioning; 5  =  severe symptoms, bed bound), and if there was any recent history (<  48 h) of an HAE attack. If patients had their laboratory investigations drawn more than 72 h after symptom onset, or if they had used rescue C1-inhibitor therapy within the preceding 7 days of their laboratory investigations being drawn, these values were excluded from the analysis.

Four patients with HAE who met inclusion criteria agreed to participate and performed baseline laboratory investigations in addition to providing clinical and laboratory data during an URTI or flu-like illness. Two of these patients provided data during a second separate illness. The results of clinical and laboratory data are summarized in Table 1. All four patients had low C4 and C1-INH function at baseline as expected. When laboratory investigations were repeated during an URTI or flu-like illness, none of the patients experienced a significant elevation of their C1-INH function into the normal range. Most patients had modest elevation of other acute phase reactants (ferritin, CRP) during their illness as compared to their baseline. Patient #2 and patient #4 had elevation of CRP above the upper limit of normal during their illnesses and also had elevation of C4 into the normal range during these infections.

**Table 1:** Laboratory investigations of patients at baseline and during an URTI or flu-like illness, along with corresponding symptom survey results

	Patient #1	Patient #2	Patient #3	Patient #4
Demographics				
Age at baseline (years)	35	69	43	67
Sex	Male	Female	Female	Female
Baseline				
Ferritin (15–247 μg/L)	103	79	**7**	**10**
CRP (< 4.8 mg/L)	0.4	2.4	< 0.3	2.1
C4 (0.09–0.50 g/L)	**0.03**	**0.07**	**0.02**	**0.07**
C1- INH function (0.70–1.30 U)	**0.16**	**0.25**	**0.12**	**0.30**
Illness #1				
Ferritin (15–247 μg/L)	121	93	**9**	49 ↑
CRP (< 4.8 mg/L)	4.2	3.6	0.3	**5.6 ↑**
C4 (0.09–0.50 g/L)	**0.05**	**0.08**	**0.04**	0.12 ↑
C1-INH function (0.70–1.30 U)	**0.22**	**0.28**	**0.14**	**0.37**
Symptoms experienced	Fever, chills, sore throat	Sore throat, rhinorrhea, nasal congestion	Fever, muscle aches, congestion, fatigue	Cough, sore throat, rhinorrhea, congestion
Duration of illness (days)	2	2	7	18
Severity of illness (1–5)	3	1	2	2
HAE attack within preceding 48 h (Y/N)	N	N	N	N
Illness #2				
Ferritin (15–247 μg/L)		123		40 ↑
CRP (< 4.8 mg/L)		**13.9 ↑**		2.5
C4 (0.09–0.50 g/L)		0.10 ↑		0.13 ↑
C1-INH function (0.70–1.30 U)		**0.24**		**0.34**
Symptoms experienced		Cough, rhinorrhea		Cough, sore throat, rhinorrhea, congestion
Duration of illness (days)		Not recorded		16
Severity of illness (1–5)		1		2
HAE attack within preceding 48 h (Y/N)		N		N

Bolded numbers are abnormal lab values

↑ Significant elevation in laboratory value from baseline (either from below the lower limit of normal to normal value, or from normal value to above the upper limit of normal)

*CRP* C-reactive protein; *C4* complement 4*; C1-INH* C1 inhibitor

## Discussion and conclusions

In the cases reviewed, we did not identify any HAE patients who had a significant change in their C1-INH functional level in the context of a mild infection. This observation suggests that C1-INH may not be a significant acute phase reactant in HAE patients. The C4 level however did increase into the normal range on three occasions (2 patients, with 1 patient having elevation during two separate illnesses). This raises the question as to the role of C4 as an acute phase reactant in HAE patients, and if it could be falsely “normal” in times of modest acute inflammation. This observation has a significant implication if C4 alone is used as a screening diagnostic test for HAE. Two of the patients did show elevation of CRP above the upper limit of normal during their illnesses, suggesting that other acute phase reactants are also activated. As none of the patients in this case series reported a very severe infection, it is unclear whether C1-INH levels would rise in the context of more significant inflammation. It is possible that a more severe pyogenic infection may alter complement levels to a larger degree. However, it should be noted that the conclusions of our case series are limited by the small number of patients who agreed to participate.

Prior studies that have looked at C1-INH as an acute phase reactant were not HAE driven studies and rather were focused on the role of C1-INH in regulating the complement pathway and modulating the inflammatory response [4–6]. In vitro studies of liver cells stimulated by interleukin-6 and TNF alpha have shown increased production of C1-INH however, the potent pro-inflammatory cytokine interleukin-1, did not increase C1-INH levels, showing that the picture is much more complicated [7]. Overall, our case series demonstrated that C1-INH does not appear to be a significant acute phase reactant in HAE patients with a mild infection and therefore, the measurement of C1-INH is likely still a reliable diagnostic marker of HAE, even in times of modest acute inflammation. C4 however can be elevated during an inflammatory state and should not be used alone as a screening test for HAE.

## Data Availability

All data generated or analysed during this study are included in this published article.

## Abbreviations

HAE: Hereditary angioedema
C1-INH: C1 inhibitor
C4: Complement 4
URTI: Upper respiratory tract infection
CRP: C reactive protein
